# Cytological Features of Pulmonary Papillary Adenoma with Malignant Transformation and Literature Review

**DOI:** 10.1155/2020/8827056

**Published:** 2020-11-17

**Authors:** Xingen Hu, Yi Chen, Guoqing Ru, Lili Yu

**Affiliations:** Department of Pathology, Zhejiang Provincial People's Hospital, People's Hospital of Hangzhou Medical College, Hangzhou 310014, China

## Abstract

Papillary adenoma of the lung is a rather rare tumor. We will present a case of papillary adenoma in the lung with malignant transformation in a 65-year-old male patient. A high dense soft tissue mass was detected in the lateral segment of the right middle lobe by CT examination. Cytologically, the tumor contained the benign cells similar to normal alveolar epithelium and the malignant cells which were significantly enlarged and irregular, crowded, or overlapping. Immunohistochemical staining showed that the epithelial cells were diffusely positive for TTF-1, napsin-A, and CK7, but were negative for p63, p40, CK5/6, CgA, Syn, CD56, and TG. The Ki67 index was about 5%. All of these evidences indicated that it was a case of papillary adenoma with malignant transformation. Thus, it should be noted that more active treatment measures should be taken to treat pulmonary papillary adenoma.

## 1. Background

Papillary adenoma of the lung is a very rare tumor. It was first reported by Spencer et al. in 1980 [1]. Up to now, there are more than 30 reported cases. In most cases, no recurrence or metastasis was found in follow-ups after biopsy or surgery. However, there are also literature reports showing that pulmonary papillary adenoma has a malignant potential behavior or malignant transformation [2–5]. At present, there are few studies on the cytological characteristics of pulmonary papillary adenoma [6, 7], and there is no report on the cytological characteristics of pulmonary papillary adenoma with malignant transformation. We will report a case of papillary adenoma with malignant transformation in the lung, observing its cytological characteristics and review the literatures.

## 2. Case Presentation

### 2.1. Clinical History

**Figure 1:**
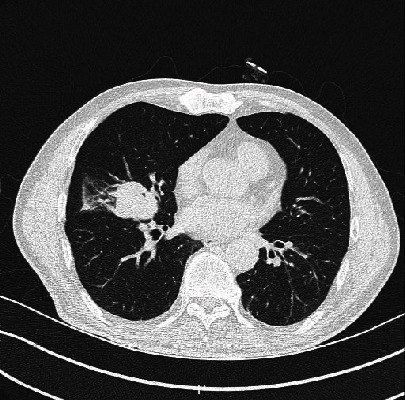
CT imaging of the chest demonstrated a density soft tissue mass in the lateral segment of the right middle lobe.

**Figure 2:**
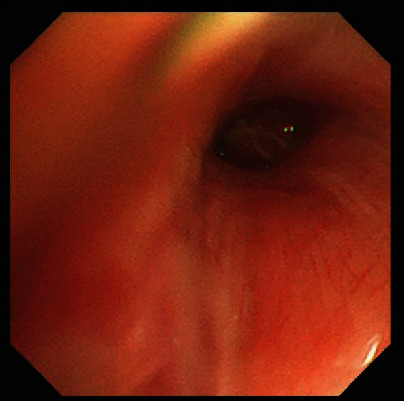
Bronchoscopy showed that the lateral branch of the right middle lobe was blocked.

**Figure 3:**
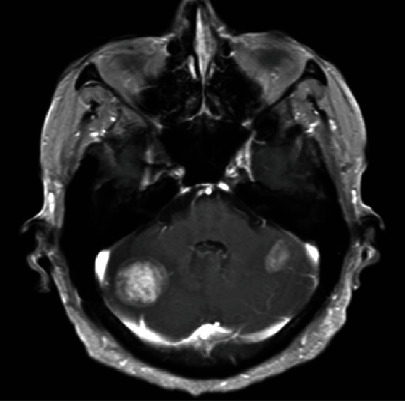
CT imaging of the head demonstrated brain metastasis.

A 65-year-old male patient came to our hospital for “dizziness for 1 week, chest CT showing right lung neoplasm, and head CT showing brain metastasis.” CT imaging of the chest showed a 42 × 34mm, in size, high dense soft tissue mass in the lateral segment of the right middle lobe (Figure 1). Bronchoscopy showed that the lateral branch of the right middle lobe was blocked (Figure 2). CT imaging of the head demonstrated brain metastasis (Figure 3). Biopsy and cytological brushing were performed.

### 2.2. Materials and Methods

The Thinprep Cytologic Test (TCT) and pap staining were used for cytology. Histological specimens were fixed with 4% neutral formaldehyde, embedded in paraffin, and sectioned 4 *μ*m thick. HE staining and immunohistochemical staining were performed. The envision two-step method was used for immunohistochemistry. Thyriod transcription factor 1 (TTF-1), napsin-A, cytokeratin 7 (CK7), P63, P40, P53, CK5/6, Ki67, chromogranin A (CgA), synaptophysin (Syn), CD56, thyroglobulin (TG), and secondary antibody were purchased from the Roche company.

## 3. Results

### 3.1. Cytopathologic Evaluation

**Figure 4:**
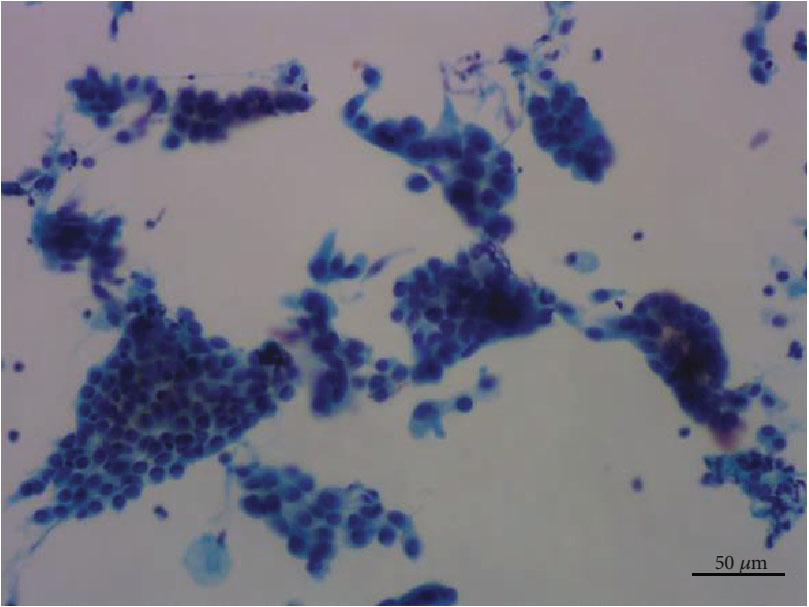
Thinprep Cytologic Test of papillary adenoma showed that the cells were arranged in flat sheets, and some cells had larger nuclei. Pap staining, ×200.

**Figure 5:**
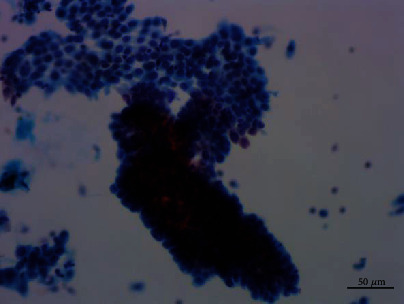
Thinprep Cytologic Test of papillary adenoma demonstrated papillary clusters. Pap staining, ×200.

**Figure 6:**
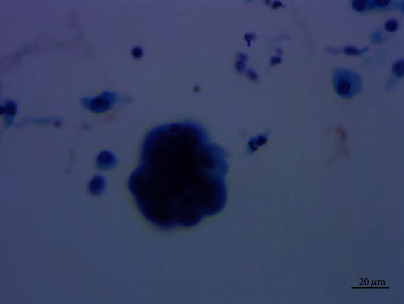
A small number of cell clusters showed three-dimensional spherical structure similar to adenocarcinoma cells. Pap staining, ×400.

**Figure 7:**
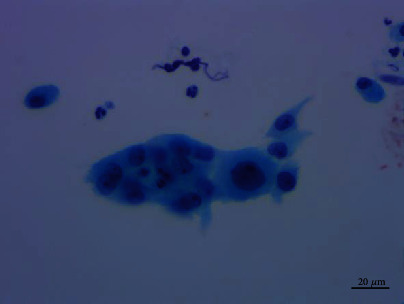
Moderate nuclear atypia cells which were 3 times the size of the contrastive cells. Pap staining, ×400.

Cytological observations under microscope showed that the cells were arranged in flat sheets/nests, papillary architecture, acini formation, and three-dimensional cluster. Most of the cells comprised of cohesive sheets of uniform cells reminiscent of type II pneumocytes or Clara cells, which were columnar or cuboidal cells with moderate amounts of cytoplasm and evenly distributed chromatin. The cells had slightly larger nuclei than those of the surrounding alveolar cells, an equal amount of eosinophilic cytoplasm, and smooth or slightly irregular nuclear membrane. Single to multiple small nucleoli were seen, and no mitotic figures or necrosis was found. The cells were streaming arrangement, similar to metaplasia or reparative cells. Some cells had more irregular nuclear membrane and crowded or overlapping nuclei with moderate cytologic atypia, which were 1.5-3 times the size of the contrastive cells with the same shape. The nucleoli were about 2-3 times the size of the contrastive cells. Some obvious eosinophilic deviated nucleoli were discovered. A small number of cells formed a three-dimensional spherical structure, similar to adenocarcinoma cells. No cilia and goblet cells were found (Figures 4–7).

### 3.2. Histopathologic Examination

**Figure 8:**
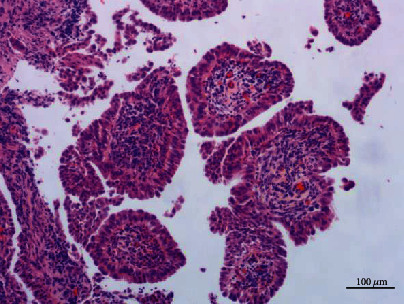
Most of the tumor cells showed simple papillary fronds with fibrovascular cores. Hematoxylin–eosin stain, ×100.

**Figure 9:**
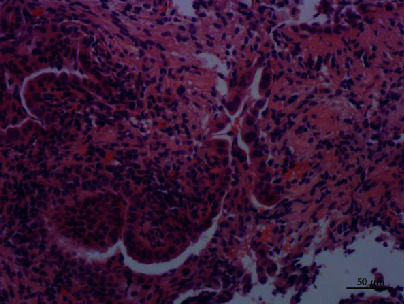
Uniform cells with evenly distributed chromatin and heteromorphic cells nearby. Hematoxylin–eosin stain, ×200.

**Figure 10:**
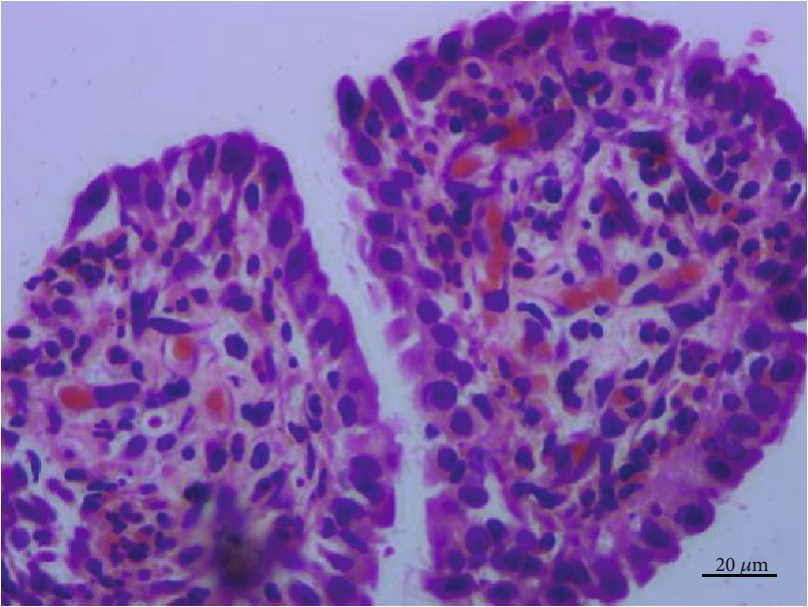
A large number of neutrophils, lymphocytes, and plasma cells infiltrated in the stroma of the fibrovascular cores. Hematoxylin–eosin stain, ×400.

**Figure 11:**
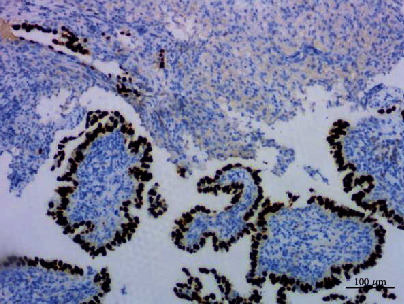
Papillary adenoma stained with TTF1, ×100.

**Figure 12:**
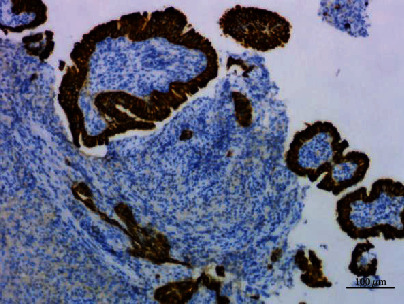
Papillary adenoma stained with CK7, ×100.

**Figure 13:**
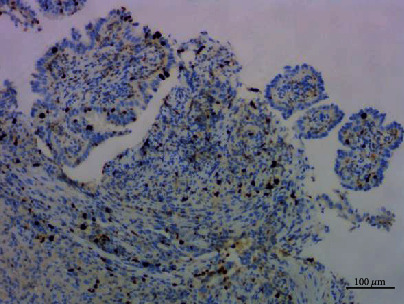
Ki67 index was about 5%, ×100.

Histologically, most of the tumor cells showed papillary fronds with fibrovascular cores. A large number of neutrophils, lymphocytes, and plasma cells infiltrated in the stroma of the fibrovascular cores. The epithelial cells were columnar or cubic and arranged in single layer. Cilia, goblet cells, and basal cells were not found. Most of the epithelial cells had minimal cytological atypia, and no mitotic figures were identified. A few cells had obvious and eosinophilic nucleoli. The cell adhesion was poor, and the cell polarity disappeared. A small number of atypical cells were arranged in a glandular pattern, and a single heterotypic cell could be seen infiltrating in the fibrotic stroma (Figures 8–10). Immunohistochemical staining showed that the tumour cells expressed TTF1, napsin A, and CK7, but not P63, P40, CK5/6, CgA, Syn, CD56, and TG. The Ki67 index was about 5% (Figures 11–13).

## 4. Discussion

Papillary adenoma of the lung is a rare tumor that mainly occurs in the peripheral part of the lung. At present, there are more than 30 cases reported in the literature, with the age spectrum ranging from 2 months to 78 years old and an average age of about 40 years. Men are slightly more common than women. The maximum diameter of the tumor is 0.4-6 cm [5]. The patients generally have no obvious clinical symptoms. Papillary adenomas are often solitary nodules with clear boundaries, usually involving only alveolar parenchyma, but there are also cases reported in the central area of the lung [8]. Under a microscope, simple papillae or partially branched papillae, and in some cases, solid or micropapillary structures can be seen [8, 9]. The papilla lined by cuboidal to columnar respiratory epithelial cells. Occasionally, ciliated cells can be seen on the surface of loose fibrous vascular stroma. The cells are mild, round, or oval, and the cytoplasm is eosinophilic or transparent. Generally, there is no cell atypia, mitotic activity, and necrosis.

Malignant transformation is the process by which cells acquire the properties of cancer. This may occur as a primary process in the normal tissue, or secondarily as malignant degeneration of a previously existing benign tumor. In our case, the tumor demonstrated a wide spectrum of cells across both benign and malignant components, and head CT showed brain metastasis. These findings indicated the process of malignant transformation.

At present, it is believed that papillary adenoma originates from primitive multipotential respiratory epithelium, which shows bidirectional differentiation into type II alveolar epithelium and Clara cells [2, 3, 10–12]. Tumor cells express CK7 and TTF-1, but do not express P40 and other basal cell markers. Pulmonary papillary tumor can occur from the bronchi to the alveoli. They have a variety of morphologies according to the location of the tumors and the presence or absence of basal cells. Papillary tumors in the bronchus include squamous cell papilloma, glandular papilloma, mixed squamous cell, and glandular papilloma. The common feature of these three types of tumors is that the epithelial components generally do not express TTF-1, and the basal cell markers such as P40 and P63 are positive. Papillary tumors in the alveoli contain papillary adenoma and papillary adenocarcinoma, which express TTF-1 but do not express the basal cell markers. In the intermediate stage, bronchiolar adenoma (including proximal type and distal type) occurs and expresses TTF-1 and the basal cell markers. From the cytological point of view, the key to distinguishing these lesions is that in our case, tumor cells showed a three-dimensional configuration, were positive for TTF-1 and napsin-A and negative for P40, but no obvious cilia and goblet cell were found. Most of the lining epithelial cells demonstrated minimal cytological atypia, and no mitotic figures were identified. No complex papillary structure, no obvious nuclear atypia, and mitosis were noted. A large number of neutrophils, lymphocytes, and plasma cells infiltrated in the stroma of the fibrovascular cores, and these differ from that of papillary adenocarcinoma. Papillary adenoma is closely related to sclerosing pneumocytoma. Sclerosing pneumocytoma is also thought to derive from primitive respiratory epithelium [13]. The difference is that papillary adenoma is arranged in a single layer on the papillary surface, and the histological morphology is simple, while the histological morphology of sclerosing pneumocytoma is diverse, and tumor cells not only arrange on the papillary surface but also in the stroma. At present, there are more and more reports that sclerosing pneumocytoma has the ability of invasion and metastasis, which is similar to papillary adenoma. Whether papillary adenoma and sclerosing pneumocytoma are the same tumor or two completely different tumors, the relationship between them needs more research to confirm. Lack of foam-like cells, intranuclear cytoplasmic inclusions, sclerosing interstitial elements, and bleeding background may contribute to the differential diagnosis of sclerosing pneumocytoma [14]. Lack of pepper-like nuclear chromatin, spindle cells, and plasma-like cells with eccentric nuclear, as well as negative for CgA and Syn, are used to differentiate papillary adenoma from lung carcinoid and atypical carcinoid. Absence of ground-glass nucleus, nuclear groove, and intranuclear pseudoinclusion bodies and negative TG immunostain can be used to differentiate from metastatic papillary thyroid carcinoma.

## 5. Conclusion

In this case, we observe the cytological features of the tumor. One part is the benign components close to the normal alveolar epithelium. The benign cells are typically mild and uniform with fine chromatin, arranged in a flat sheet pattern. The other part is the malignant components. The malignant cells are significantly enlarged and irregular, crowded, or overlapping. The nucleoli are eosinophilic. Some cells formed a three-dimensional spherical structure. Histologically, the benign components present a simple papillary structure, without branching papillae or budding. The malignant components show glandular arrangement or infiltrative growth of tumor cells in the fibrous stroma. All of these evidences indicate that this is a case of papillary adenoma with malignant transformation. From this case, we not only have a profound understanding of the cytological characteristics of pulmonary papillary adenoma with malignant transformation but also obtain important guiding information for clinical practice. As to pulmonary papillary adenoma, we should take more active treatment measures, and clinical follow-up observation should be performed.

## Data Availability

All data generated or analyzed during this study are included in this article.
